# Interferon regulatory factor 3 mediates effective antiviral responses to human coronavirus 229E and OC43 infection

**DOI:** 10.3389/fimmu.2023.930086

**Published:** 2023-05-01

**Authors:** Joseph K. Sampson Duncan, Danyang Xu, Maria Licursi, Michael A. Joyce, Holly A. Saffran, Kaiwen Liu, Jin Gohda, D. Lorne Tyrrell, Yasushi Kawaguchi, Kensuke Hirasawa

**Affiliations:** ^1^ Division of BioMedical Sciences, Faculty of Medicine, Memorial University of Newfoundland, St. John’s, NL, Canada; ^2^ Li Ka Shing Institute of Virology, University of Alberta, Edmonton, AB, Canada; ^3^ Department of Medical Microbiology and Immunology, University of Alberta, Edmonton, AB, Canada; ^4^ Research Center for Asian Infectious Diseases, The Institute of Medical Science, The University of Tokyo, Tokyo, Japan; ^5^ Division of Molecular Virology, Department of Microbiology and Immunology, The Institute of Medical Science, The University of Tokyo, Tokyo, Japan; ^6^ Department of Infectious Disease Control, International Research Center for Infectious Diseases, The Institute of Medical Science, The University of Tokyo, Tokyo, Japan

**Keywords:** SARS-CoV2, OC43, 229E, innate immunity, interferon, IRF1, IRF3, IRF7

## Abstract

Interferon regulatory factors (IRFs) are key elements of antiviral innate responses that regulate the transcription of interferons (IFNs) and IFN-stimulated genes (ISGs). While the sensitivity of human coronaviruses to IFNs has been characterized, antiviral roles of IRFs during human coronavirus infection are not fully understood. Type I or II IFN treatment protected MRC5 cells from human coronavirus 229E infection, but not OC43. Cells infected with 229E or OC43 upregulated ISGs, indicating that antiviral transcription is not suppressed. Antiviral IRFs, IRF1, IRF3 and IRF7, were activated in cells infected with 229E, OC43 or severe acute respiratory syndrome-associated coronavirus 2 (SARS-CoV-2). RNAi knockdown and overexpression of IRFs demonstrated that IRF1 and IRF3 have antiviral properties against OC43, while IRF3 and IRF7 are effective in restricting 229E infection. IRF3 activation effectively promotes transcription of antiviral genes during OC43 or 229E infection. Our study suggests that IRFs may be effective antiviral regulators against human coronavirus infection.

## Introduction

Human coronaviruses are enveloped single-stranded RNA viruses with positive-sense genomes that commonly cause respiratory tract infection in humans (1, 2). They are comprised of 4 genera: alphacoronavirus, betacoronavirus, gammacoronavirus, and deltacoronavirus. Certain betacoronaviruses are known to cause lethal infection in humans, including middle east respiratory syndrome (MERS), severe acute respiratory syndrome-associated coronavirus (SARS-CoV) and SARS-CoV-2. MERS infection was first found in 2012; since then, 2249 infections and 858 deaths in 27 countries have been reported (3). SARS-CoV caused 8237 infections and 775 deaths in more than 30 countries in 2002 (4). SARS-CoV-2 was identified in 2019 and is responsible for the current COVID-19 pandemic. As of today (March 1, 2023), 679 million cases and 6.8 million deaths have been reported worldwide (5). Other human coronaviruses, such as OC43, 229E, NL63 and HKUI1, infect the upper respiratory tract and cause common seasonal cold symptoms (6). OC43 and HKUI1 are members of the genera betacoronaviruses, while 229E and NL63 are alphacoronaviruses (7, 8). As the sequence of non-structural proteins are well-conserved among human coronaviruses, they share very similar replication cycles (9, 10).

Cells sense viral products intracellularly and extracellularly using different pattern recognition receptors (PRRs) such as toll-like receptors, RIG-I-like receptors and melanoma differentiation-associated gene 5 (MDA-5) (11). The recognition of viral products results in activation and nuclear translocation of IFN regulatory factor 3 (IRF3), IFN regulatory factor 7 (IRF7) and nuclear factor-κB (NF-κB), which activate the transcription of interferons (IFNs) (12, 13). IFNs, which have three classes, type I (IFN-α/β), type II (IFN-γ) and type III (IFN-λ) IFNs, play essential roles in antiviral innate immune response (14, 15). Secreted IFNs bind to IFN receptors in an autocrine or paracrine manner and activate the Janus kinase (JAK)-signal transducer and activator of transcription (STAT) (16, 17). Phosphorylated STAT proteins along with other transcriptional regulators such as IRF1 and IRF9 directly bind to the promoter regions of IFN-stimulated genes (ISGs) to activate their transcription (18, 19). Human coronaviruses are generally sensitive to antiviral functions of IFNs albeit with some differences in their sensitivity. Both SARS-CoV and MERS are sensitive to IFN when cells are treated at high concentrations (20–23). Between the two viruses, IFNs are more effective in inhibiting the replication of MERS than SARS-CoV (23). Moreover, SARS-CoV-2 is more sensitive to type I IFNs than SARS-CoV (24, 25). As for other human coronaviruses, IFNs suppress OC43 infection in a cell type dependent manner (26), while 229E is sensitive to IFN treatment *in vitro (* 20, 27). These reports suggest that human coronaviruses are generally sensitive to IFNs, but each virus has different levels of sensitivity.

In clinical settings, IFN-β treatment significantly reduced the mortality of SARS-CoV-2 infected patients when administrated at an early stage of infection (28). Similarly, treatment of pegylated IFN-α significantly reduced viral replication of SARS-CoV in macaques (29). In STAT1 -/- mice, SARS-CoV induced a prolonged infection with higher viral loads in the lung, suggesting that the JAK/STAT pathway downstream of IFN receptors is essential for clearing SARS-CoV *in vivo (* 30). However, SARS-CoV infection was not exacerbated in IFN-α/β receptor -/- mice, but instead mouse survival was improved due to reduced immune cell infiltration in the lung, indicating immunopathogenic roles of IFNs in SARS-CoV infection (31).

While the antiviral efficacy of IFNs against human coronavirus is clear, SARS-CoV-2 infected patients displayed low production of type I and III IFN and a moderate ISG response (32). Similarly, type I IFN response was delayed in mice infected with SARS-CoV-2, allowing viral replication, lung immunopathogenesis and lethal pneumonia (31). These reports suggest that IFN-mediated antiviral innate responses are dysregulated in SARS-CoV-2 infection *in vivo*. This is most likely due to the presence of SARS-CoV-2 proteins that suppress the production of IFNs and IGSs (33). In summary, IFNs have antiviral and immunopathogenic roles in human coronavirus infection. Moreover, IFN antiviral responses are targets of immune evasion mechanisms by human coronaviruses.

Among IRFs, IRF1, IRF3, and IRF7 are transcriptional regulators of IFNs and ISGs (34, 35). IRF1 is upregulated during viral infection or IFN stimulation, which, in turn, activates transcription of type I IFNs (36, 37). As IRF1 is a co-transcriptional factor of ISGs regulated by the JAK/STAT pathway, a subset of ISGs can be induced by IRF1 in an IFN-independent manner (38). Upon virus infection, the innate immune sensors interact with viral components and activate the TANK-binding Kinase 1 (TBK)/κB kinase ϵ (IKKϵ) complex, which induces the activation of both IRF3 and IRF7 (39, 40). The activation of IRF3 and IRF7 results in the translocation of these proteins to the nucleus where they initiate the transcription of type I IFNs. Similar to IRF1, IRF3 can also exhibit antiviral functions independently of the IFN system by upregulating ISGs independently of IFN production *in vitro* (41, 42). IRF3 could be an important component of innate immune responses against SARS-CoV-2, as blocking phosphorylation and translocation of IRF3 promotes its replication (43). Accumulating evidence suggests that human coronaviruses can interfere with the activity of IRF3. SARS-CoV-2 PLpro and 3Clpro, viral proteins responsible for cleaving viral polyproteins, also degrade IRF3 (44, 45). Other studies demonstrated that SARS-CoV-2 7a reduces IRF3 phosphorylation by downregulating TBK1 expression levels (46, 47). Similarly, SARS-CoV 8b and 8ab induce IRF3 degradation in a ubiquitin dependent manner (44), while MERS M protein disrupts the interaction of TNF Receptor Associated Factor 3 (TRAF3) and TBK1, leading to reduced IRF3 activation (44). These studies clearly suggest that human coronavirus proteins target IRF3 to promote their replication. In contrast to IRF3, antiviral roles of IRF1 and IRF7 against human coronavirus infection are less understood. In animal coronaviruses, IRF1 was shown to have antiviral properties against mouse hepatitis virus (MHV) (48). The viral M protein of porcine epidemic diarrhea virus (PEDV) interacts with IRF7 and inhibits its antiviral functions (33, 49). Thus, it is possible that IRF1 and IRF7 also have antiviral effects in human coronavirus infection.

Although it is suggested that the antiviral IRFs are important for host antiviral responses against human coronavirus infection, there is no direct functional evidence reported. In this study, we conducted loss- and gain-of-function experiments of IRF1, IRF3 and IRF7 to clarify antiviral functions of IRF1, IRF3 and IRF7 during human coronavirus infection. To fight against human coronavirus infection, it is important to gain more knowledge about antiviral responses mediated by IRFs during human coronavirus infection.

## Materials and methods

### Cells, viruses, and reagents

Human lung fibroblast cell line MRC5, human lung cancer cell line H1299, monkey kidney epithelial cell line Vero E6, mouse fibroblast cell line L929, human coronaviruses HcoV-OC43 and HcoV-229E were obtained from the American Type Culture Collection (ATCC; Manassas, VA, USA). Human dermal fibroblast cells were obtained from Cell Applications Inc. (San Diego, CA, USA). Vesicular stomatitis virus (VSV, Indiana strain) was provided by Dr. John C. Bell (Centre for Innovative Cancer Therapeutics, Ottawa Hospital Research Institute, Ottawa, Canada). VSV was amplified and titrated by plaque assay using L929 cells as described previously (50). SARS-COV-2 (SARS-CoV-2/CANADA/VIDO/01/2020) was isolated at the VIDO, University of Saskatchewan from a clinical specimen obtained at the Sunnybrook Health Sciences Centre, and propagated at the National Microbiology Laboratory (NML) was amplified and titrated by plaque assay using Vero E6 cells (51). Recombinant human IFN-α2 A, human IFN-γ and IFN-λ 1 were obtained from Bio-Rad, BD Pharmingen and R&D Systems respectively. Antibodies used in this study include: IRF3, phospho-IRF3, IRF7, phsopho-STAT1 (Cell signalling technology), IRF3 (Santa Cruz), IRF1 (BD Transduction Laboratories), GAPDH (Santa Cruz Biotechnology), 229E N protein (Ingenasa), OC43 N protein (Millipore), SARS-CoV-2 spike protein (Sino Biological). Negative control siRNA, IRF1 siRNA (s7501), IRF3 siRNAs (s7509) and IRF7 siRNA (s223948) were purchased from Life Technologies. IRF1-pINCY plasmid (Open biosystems) and IRF7 -ORF vector (Applied Biological Materials) was subcloned into pcDNA3 plasmid (Addgene). pcDNA3-IRF3 was purchased from Addgene.

### Cell culture

All cells were cultured in high-glucose Dulbecco’s modified Eagle’s medium (Corning, MA) supplemented with 10% fetal bovine serum (HyClone, Cytiva), 1 mM sodium pyruvate (Life Technologies) and antibiotic-antimycotic (Thermo Scientific). Cells were maintained in 10-cm culture dishes at 37°C with 5% CO_2_ for the use of experiments in this work. Hunan Dermal Fibroblasts were grown on dishes coated with 0.1% gelatine (from Cell Applications, Inc).

### Virus infection

Cells with 90% confluency were infected with human coronavirus 229E or OC43 with a MOI of 0.01. The diluted stock viruses were adsorbed for 2 hours at 33°C, and then removed and replaced with DMEM with 2% FBS. Infected cells were incubated at 33°C with 5% CO_2_ for up to six days. For IFN treatment, MRC5 cells were treated with IFN-α (250 and 500 U/ml), IFN-γ (50 and 100 U/ml) or left untreated for 18 hours and then challenged with 229E or OC43. For siRNA knockdown, cells were transfected with 5 pmol siRNA oligos using Lipofectamine RNAiMAX Transfection Reagent (Life Technologies) and 24 hours later challenged with or without 229E or OC43 (MOI of 0.01). For overexpression of IRFs, cells in 24-well plates (4×10^4^ cells/well) were transfected with control pcDNA3, IRF1-pcDNA3, IRF3-pcDNA3 OR IRF7-pcDNA3 using Lipofectamine 3000 Transfection Reagent (Life Technologies) and 24 hours later challenged with or without 229E or OC43. VSV absorption and infection (MOI of 0.0001) were conducted at 37°C with 5% CO_2_. The amount of progeny viruses in the culture supernatant was measured by TCID50 (50% tissue culture infective dose) assay for 229E and OC43 (52) and plaque assay for VSV (50).

Vero E6 cells expressing TMPRSS2 were seeded into 12 well plates and infected with SARS CoV-2 at MOI 0.01 or 0.1 and absorbed for 1 hour at 37°C in DMEM with 2% FBS. After absorption period, virus was aspirated and replaced with DMEM containing 5% FBS. At 8 or 24 hours post infection (hpi), cells were washed with PBS and harvested with RIPA buffer. All infections were performed in the Canada Foundation for Innovation Containment Level 3 Facility at the University of Alberta.

UV-inactivation of OC43 and 229E was performed by diluting the virus in 4ml DMEM with 2% FBS and placed in a 6 cm plastic petri dish at 45 cm from a 60 W ultraviolet tube for 10 min (53).

### Western blot analysis

Cells in each well of a 24-well plate were harvested with 100 μl radioimmunoprecipitation assay (RIPA) buffer supplemented with protease inhibitors (Sigma-Aldrich) and phosphatase inhibitors (Thermo Scientific). SDS sample buffer was added to cell lysates, followed by a 5-minute boiling period. The same volume of each sample was subjected to 10% SDS-PAGE. To use the housekeeping protein (GAPDH) as an indicator of infection/cell death in the experiments, the loading amounts of the samples were not adjusted by protein assay. The proteins were then transferred to nitrocellulose membranes (Bio-Rad, ON, Canada) using a Trans-blot Turbo Transfer System (Bio-Rad). The membrane was blocked with 5% skim milk in tris-buffered saline (TBS) with 0.1% Tween 20 (TBS-T) for 1 hour at room temperature and then incubated with primary antibodies overnight at 4°C. The following day, membranes were incubated with peroxide-conjugated anti-rabbit or anti-mouse secondary antibody (Santa Cruz Biotechnology) for 1 hour. Specific bands were detected with ImageQuant LAS 4000 (GE Healthcare Life Sciences, QC, Canada) using enhanced chemiluminescence western blotting detection reagent (Bio-Rad or Amersham).

### Quantitative RT-PCR

Quantitative RT-PCR (RT-qPCR) was performed in triplicate using the previously described validation strategies (54). RNA was isolated from MRC5 and H1299 cells using TRIzol (Invitrogen) according to the manufacturer’s instructions. cDNA was synthesized from the RNA using the RevertAid H Minus First Strand cDNA Synthesis Kit (Thermo Scientific). Quantitative PCR (qPCR) was performed in triplicates using powerSYBR^®^Green PCR Master Mix (Life Technologies LTD, UK) and analyzed with StepOnePlus qPCR system (Applied Biosystems, CA). The polymerase chain reaction (PCR) procedure was as manufacturer’s instructions: 95°C for 10 minutes followed by 40 cycles of 95°C for 15 seconds, 60°C for 1 minute, and then followed by melt-curve analysis. For data analysis, mRNA levels of each gene were normalized to GAPDH. The fold change of each sample toward the parental cells sample was calculated using the 2^−ΔΔCT^ method. The experiment was conducted with biological triplicates. The primer sequences are shown in **Supplementary Table 1**. A five-point, five-fold dilution series was used for primer validation.

### Statistical analysis

One-way ANOVA and two-way ANOVA with Dunnett’s or Turkey’s *post-hoc* test were performed using GraphPad Prism 6.0 software.

## Results

### IFN treatment delays 229E infection but not OC43 infection

**Figure 1:**
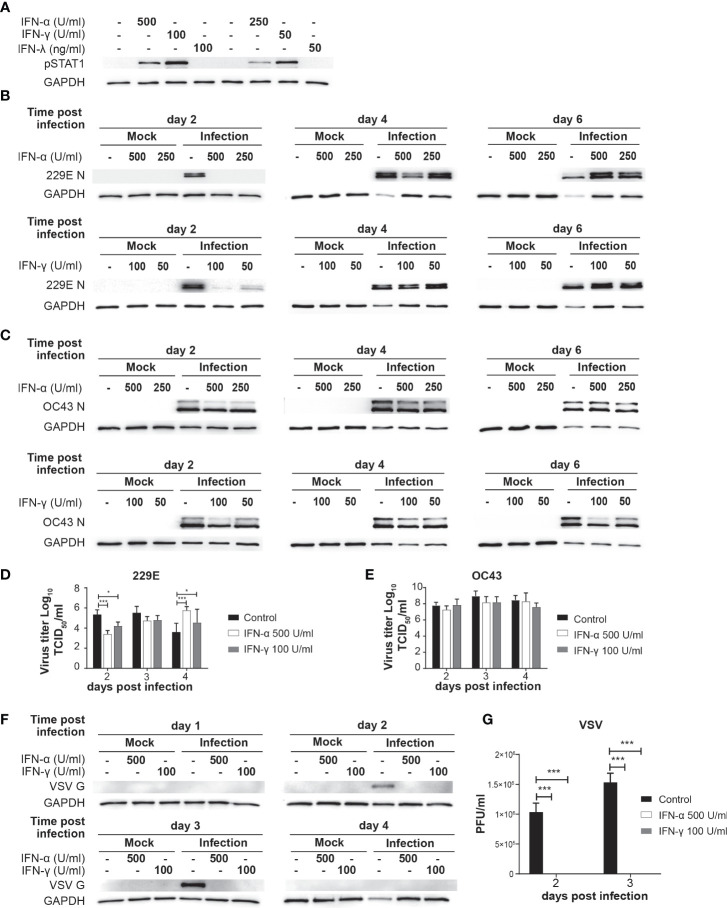
IFN treatment delays 229E but not OC43 infection. **(A)** MRC5 cell were left untreated or treated with IFN-α (500 and 250 U/ml), IFN-γ (100 and 50 U/ml) or IFN-λ (100 and 50 ng/ml) for 30 min. STAT1 activation was determined by western blot analysis using anti phospho-STAT1 and GAPDH antibodies. **(B, C)** MRC5 cells were left untreated or treated with IFN-α or IFN-γ for 18 hours, and then challenged with 229E **(B)** or OC43 **(C)** infection at MOI of 0.01. Western blot analysis of viral protein was conducted using anti 229E N protein **(B)**, OC43 N protein **(C)** and GAPDH antibodies. **(D, E)** TCID_50_ assay was performed to measure the progeny virus of 229E or OC43 infected MRC5 cells left untreated or treated with IFNs (n=3). **(F, G)** MRC5 cells were left untreated or treated with IFN-α (500U/ml) or IFN-γ (100U/ml), and then challenged with VSV at MOI of 0.0001. VSV infection was determined by **(F)** western blot analysis using anti VSV G and GAPDH antibodies and **(G)** plaque assay using L929 cells (n=3). The amount of progeny virus is shown as plaque forming units (PFU)/ml of samples nontreated or treated with IFN. *p<0.05, ***p<0.001, Two-way ANOVA.

To investigate the antiviral effects of IFNs on human coronavirus infection, we first tested if human lung fibroblast cells, MRC5, are sensitive to different types of IFNs (**Figure 1A**). When MRC5 cells were treated with IFN-α, IFN-γ or IFN-λ for 30 mins, we observed STAT1 phosphorylation in cells treated with IFN-α or IFN-γ, but not in those treated with IFN-λ. This indicates that MRC5 cells are sensitive to type I and type II IFN-λ, but not to type III IFN. MRC5 cells are reported to lack IFN-Lambda; receptor (55, 56). Therefore, we focused on type I and II IFN for the following experiments.

MRC5 cells were left untreated or treated with IFN-α (250 and 500 U/ml) or IFN-γ (50 and 100 U/ml) for 18 hours and then challenged with 229E (**Figure 1B**) or OC43 (**Figure 1C**) at a MOI of 0.01. Cell lysates were harvested at 2, 4 and 6 days after infection for western blot analysis of viral nucleocapsid proteins N and GAPDH. At 2 days following 229E infection, viral protein was detected in cells without IFN-α treatment, but not in cells treated with IFN-α (**Figure 1B**). At 4 days after infection, less viral protein was detected with a higher IFN-α concentration (500 U/ml). Similarly, IFN-γ treatment inhibited 229E infection at 2 days, but not at 4 and 6 days after infection. In contrast, OC43 infection was not significantly affected by IFN-α or IFN-γ, although some minor reductions in viral protein levels were observed in cells treated with IFNs at 2 days after infection (**Figure 1C**). To further confirm the effect of IFNs on virus production, we conducted a progeny virus assay (**Figures 1D, E**). The amount of progeny 229E was significantly lower in cells treated with IFN-α or IFN-γ at 2 days after infection (**Figure 1D**). On the other hand, IFN treatment did not reduce the progeny virus production of OC43 (**Figure 1E**). Similarly, OC43 infection was not sensitive to IFN treatment in human lung cancer cell line H1299, while 229E infection was inhibited (**Supplementary Figure 1**). To confirm the efficacy of IFN to induce sufficient antiviral responses, we conducted a positive control experiment where MRC5 cells were treated with the same amount of IFN-α or IFN-γ, and then challenged with an IFN-sensitive virus, vesicular stomatitis virus (VSV). The IFN treatment completely inhibited VSV protein synthesis (**Figure 1F**) and progeny virus production (**Figure 1G**), indicating the concentration of IFN used in our system is sufficient to inhibit the replication of an IFN-sensitive virus. At 4 days after VSV infection, the expression levels of GAPDH and VSV G were very low in infected cells without IFN treatment, indicating that VSV replication was not active because most cells were dead.

Taken together, these results suggest that both type I and II IFN delay 229E infection in MRC5 and H1299 cells, but they are not effective in protecting against OC43 infection.

### 229E and OC43 infection activate transcription of IFN-stimulated genes

**Figure 2:**
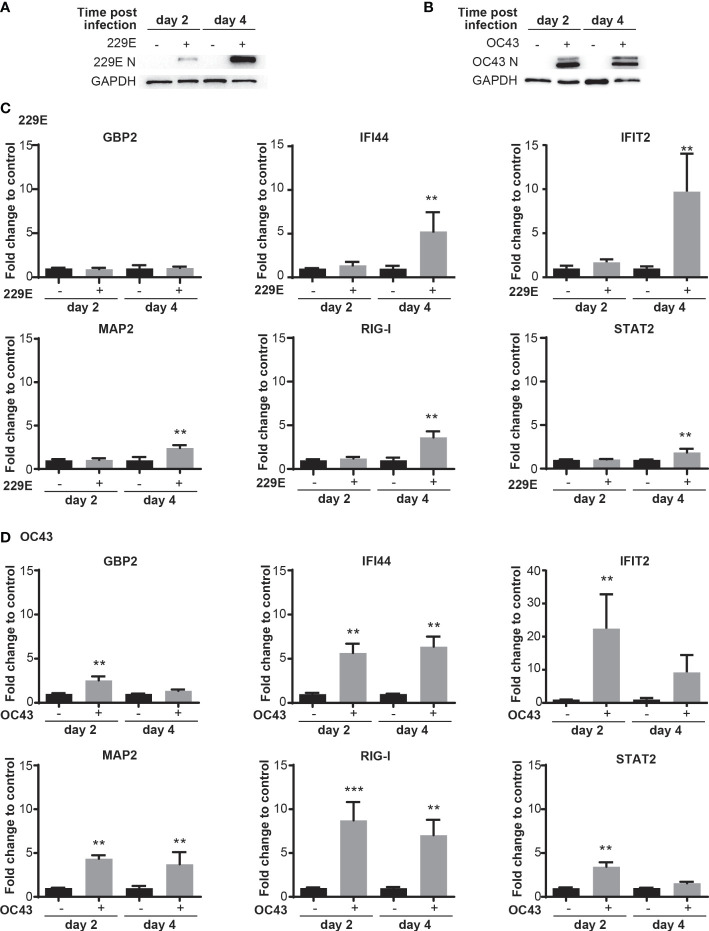
Human coronavirus infection activates transcription of IFN-stimulated genes. MRC5 cells were left uninfected or infected with 229E **(A, C)** or OC43 **(B, D)** at MOI of 0.01. Infection of 229E **(A)** and OC43 **(B)** was confirmed by western blot analysis using anti 229E N protein, OC43 N protein and GAPDH antibodies. The mRNA levels of ISGs (GBP2, IFI44, IFIT2, MAP2, RIG-I and STAT2) were determined by RT-qPCR in MRC5 cells infected with 229E **(C)** or OC43 **(D)** (n=6). The fold change to control indicates the fold change of the expression level for infected samples towards that of the non-infected controls at the same time point post infection. The transcriptional level for each gene was calculated by normalizing to GAPDH expression level and then normalized by the corresponding control. **p<0.01, ***p<0.001 for RIG-I OC43, Two-way ANOVA.

Our next question was whether human coronaviruses activate antiviral innate responses in infected cells. To test this, we assessed the transcriptional activation of ISGs at 2 and 4 days after human coronavirus infection (**Figure 2**). Western blot analysis was first conducted to confirm infection of 229E (**Figure 2A**) and OC43 (**Figure 2B**). Then, the expression of the following IFN-inducible genes was examined during 229E (**Figure 2C**) or OC43 (**Figure 2D**) infection: guanylate binding protein 2 (GBP2) (57), interferon induced protein 44 (IFI44) (58), interferon induced protein with tetratricopeptide repeats 2 (IFIT2) (59), microtubule-associated protein 2 (MAP2) (54), retinoic acid-inducible gene I (RIG-I) (60) and STAT2 (61). 229E infection did not induce GBP2, but significantly increased the expression of all other genes at 4 days after infection. In contrast, OC43 infection induced all IFN-inducible genes tested at 2 days after infection, and the expression levels were significantly higher than control at 4 days except for GBP2 and STAT2. The expression of the ISGs was induced at the earlier stage of infection in cells infected with OC43 than those infected with 229E. The ISG induction was not observed in cells infected with UV-inactivated 229E or OC43 (MOI of 0.01), suggesting that active infection is required for the ISG induction (**Supplementary Figure 2**). While GBP2 induction in MRC5 cells infected with 229E or OC43 infection was lower than those stimulated with IFN-γ, the expression levels of RIG-I and STAT2 induced by 229E or OC43 infection were similar to those induced by IFN-α or -γ stimulation (**Supplementary Figure 2**). These results demonstrate that human coronavirus infection induces host antiviral transcriptional responses.

### IRF1, IRF3 and IRF7 are activated during human coronavirus infection

**Figure 3:**
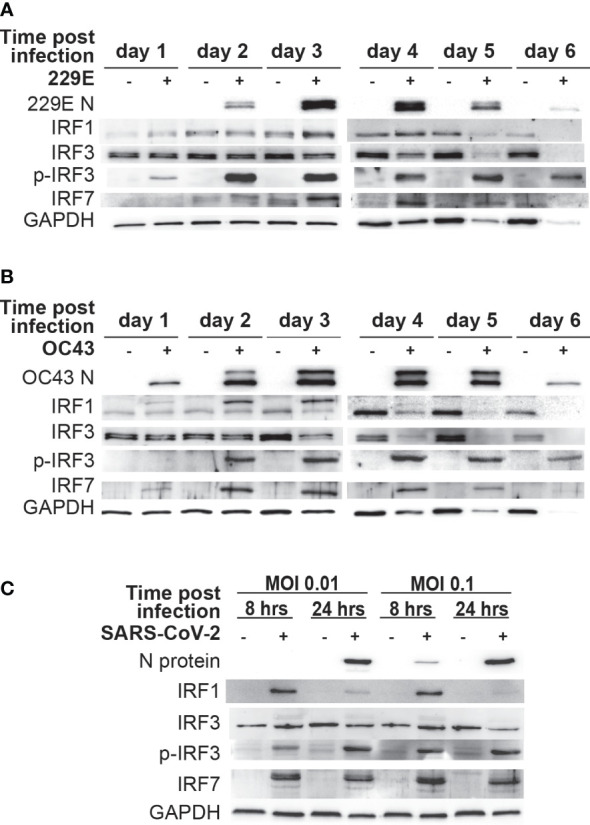
Human coronavirus infection activates IRF1, IRF3 and IRF7. MRC5 cells were left uninfected (-) or infected (+) with 229E **(A)** or OC43 **(B)** at MOI of 0.01. **(C)** Vero E6 cells expressing TMPRSS2 were left uninfected or infected with SARS-CoV-2 at MOI of 0.01 or at MOI 0.1. The activation of IRF1, IRF3 and IRF7 was determined by western blot analysis using antibodies against 229E N protein, OC43 N protein, SARS-CoV-2 N protein, IRF1, IRF3, IRF7, phosphorylated (p-IRF3) and GAPDH.

As IRF1, IRF3 and IRF7 are the key transcriptional regulators of IFNs and ISGs, we sought to determine their activation status during human coronavirus infection. Accordingly, a western blot analysis was conducted to assess the expression of IRF1 and IRF7, and phosphorylation of IRF3 (an active form of IRF3) in MRC5 cells infected with 229E or OC43. After 229E infection, viral proteins were detected from day 2 and reached a peak at day 3 and 4 (**Figure 3A**). The expression of IRF1 and IRF7 was increased at 3 and 4 days after infection compared to uninfected controls. Similarly, phosphorylated IRF3 increased at the same time points. After OC43 infection, OC43 nucleoprotein was detected at day 1, which peaked at 3 and 4 days after infection (**Figure 3B**). In these infected cells, IRF1 expression increased at day 2 and 3. We observed an upper shift in IRF1 bands, which could be caused by posttranslational modifications of IRF1. IRF3 and IRF7 were also activated from 2 to 5 days after OC43 infection. Activation of IRFs was not observed in cells infected with UV-inactivated 229E or OC43 infection while IFN stimulation induces higher activation of the IRFs than 229E or OC43 infection (**Supplementary Figure 3**). We also confirmed the activation of IRF1 and IRF3 in human primary dermal cells (**Supplementary Figure 4**). The dermal cells supported 229E or OC43 infection, which increased IRF1 expression at day 2 and 3, and phosphorylation of IRF3 from day 2 to 5. However, activation of IRF7 was not observed.

To determine the effect of SARS-CoV-2 infection on IRFs, Vero E6 cells expressing TMPRSS2 were infected with SARS-CoV-2 a MOI of 0.01 or 0.1 (**Figure 3D**). Viral proteins were detected in cells infected with a MOI of 0.01 at 24 hours post-infection, and at 8 and 24 hours post-infection with infection at a MOI of 0.1. In SARS-CoV-2 infected cells, there was an increase in IRF1 and IRF7 expression and IRF3 phosphorylation, suggesting that SARS-CoV-2 infection also activates antiviral IRFs.

These results demonstrate that IRF1, IRF3 and IRF7 are activated during human coronavirus infection.

### IRF1, 3 and 7 have antiviral roles against human coronavirus infection

**Figure 4:**
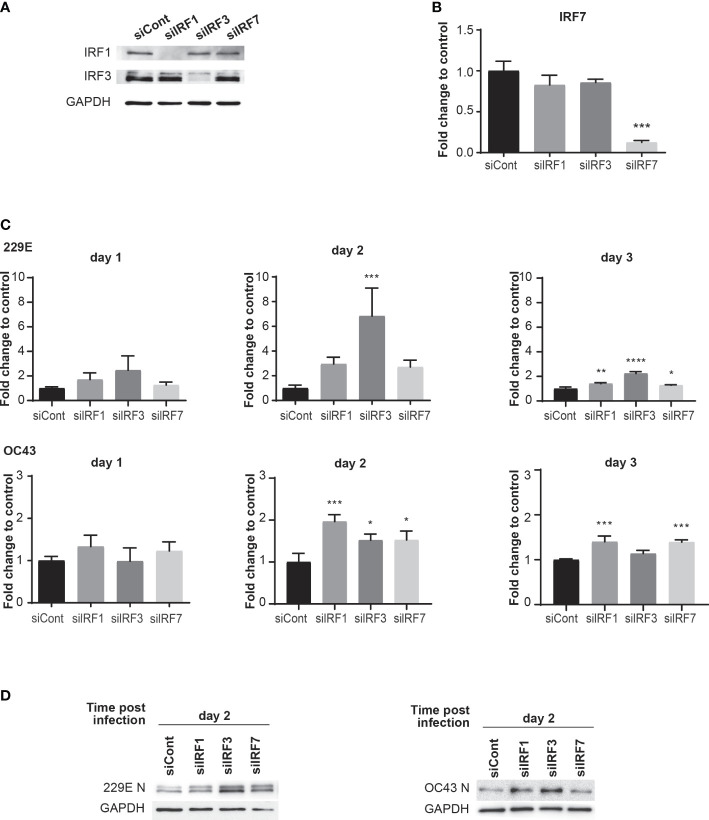
Knockdown of IRFs promotes human coronavirus infection. MRC5 cells were transfected with control siRNA (siCont), IRF1 siRNA, IRF3 siRNA or IRF7 siRNA oligoes (5 pmol) using Lipofectamine RNAiMAX transfection reagent. The knockdown of IRFs expression was confirmed by western blot analysis for IRF1 and IRF3 **(A)** and RT-qPCR for IRF7 **(B)**. MRC5 cells were then infected with 229E or OC43 at MOI of 0.01. **(C)** The amounts of viral RNA were measured by RT-qPCR (n=3). **(D)** The amounts of viral protein were determined by western blot analysis using antibodies against 229E N protein, OC43 N protein and GAPDH. For RT-qPCR analysis, the transcription level for each gene was first normalized to GAPDH expression level. The fold change to control indicates the fold change of the expression level for IRFs siRNA transfected samples to that of the control siRNA transfected samples. *p<0.05, **p<0.01, ***p<0.001, ****p<0.0001, Two-way ANOVA.

To investigate the functional roles of IRF1, IRF3 and IRF7 during human coronavirus infection, we conducted a loss-of-function analysis using siRNA knockdown. MRC5 cells were transfected with either control siRNA oligos or those against IRF1, IRF3 or IRF7 for 24 hours. The knockdown of IRF1 and IRF3 was confirmed with western blot analysis, which showed lower expression levels in cells treated with their corresponding siRNA oligos (**Figure 4A**). As IRF7 expression is undetectable by western blot in non-infected cells, IRF7 knockdown was confirmed by qPCR analysis (**Figure 4B**). When these cells were challenged with 229E or OC43, RT-q-PCR analysis revealed that IRF1 knockdown promotes 229E infection at 3 days after infection and OC43 infection at 2 and 3 days after infection (**Figure 4C**). IRF3 knockdown also increased the expression of viral RNA at 2 and 3 days after 229E infection and 2 days after OC43 infection. In addition, IRF7 knockdown resulted in an increase in 229E infection at 3 days after infection and OC43 infection at 2 and 3 days after infection. These results were further confirmed by western blot analysis (**Figure 4D**). 229E viral protein synthesis was increased in cells treated with siRNA oligos against IRF3 or IRF7 compared to siRNA controls at 2 days after infection. For OC43 infection, knockdown of IRF1 or IRF3 promoted viral protein synthesis compared to siRNA controls at both 2 and 3 days after infection. Altogether, these loss-of-function experiments indicate the antiviral roles of IRF1, IRF3 and IRF7 against coronavirus infection.

**Figure 5:**
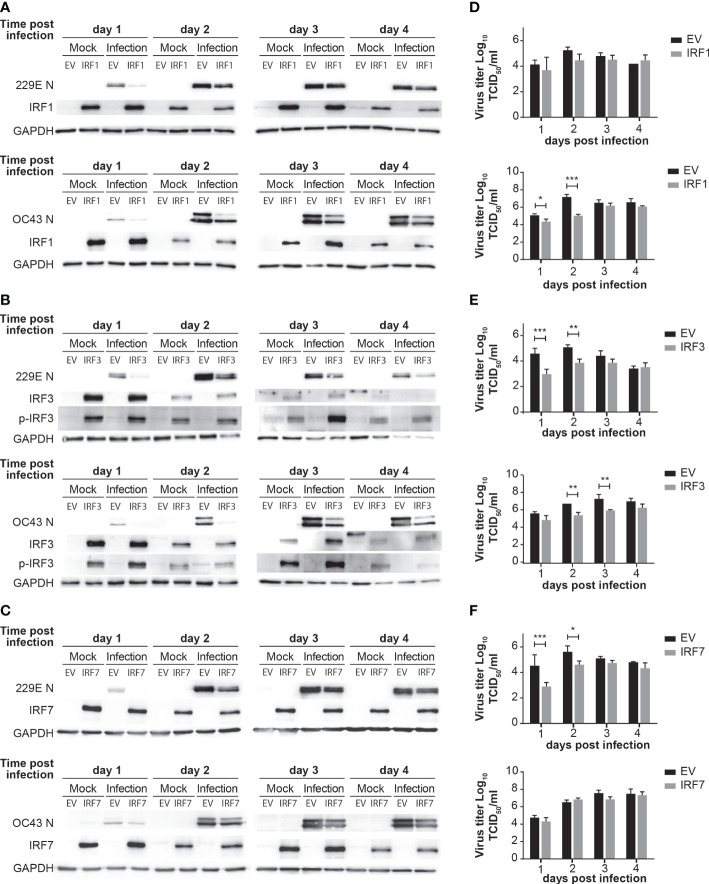
Overexpression of IRFs inhibits human coronavirus infection. H1299 cells were transfected with control pcDNA3 empty plasmid (EV) or the plasmid containing IRF1 **(A)**, IRF3 **(B)** or IRF7 **(C)** and then infected with or without 229E or OC43 at MOI of 0.01. Virus infection was determined by western blot analysis using antibodies against 229E N protein, OC43 N protein, IRF1, IRF3, IRF7, phosphorylated IRF3 (p-IRF3) and GAPDH. Amounts of progeny viruses were measured in the supernatant of cells transfected with the plasmid containing IRF1 **(D)**, IRF3 **(E)** or IRF7 **(F)** by TCID_50_ assay which were compared to those transfected with empty plasmid (n=6). *p<0.05, **p<0.01, ***p<0.001, Two-way ANOVA.

To further confirm the antiviral roles of IRF1, IRF3 and IRF7, we conducted gain-of-function experiments *via* overexpression. H1299 cells were transfected with control pcDNA3, IRF1-pcDNA3, IRF3-pcDNA3 or IRF7-pcDNA3 for 24 hours and then challenged with 229E or OC43 (**Figure 5**). IRF1 overexpression effectively inhibited replication of OC43 as the generation of viral proteins and progeny viruses were lower in cells transfected with IRF1-pcDNA3 than those transfected with control pcDNA3 (**Figure 5A**). We observed a slight reduction of 229E N protein expression in IRF1 overexpressed cells at 1 day after infection, but there was no significant difference in the amount of progeny virus (**Figure 5D**). Moreover, 229E and OC43 generated less viral proteins and progeny viruses in the cells transfected with IRF3-pcDNA3 (**Figures 5B, E**), suggesting that IRF3 introduction promoted antiviral activities against both 229E and OC43. Finally, the introduction of IRF7 effectively reduced 229E infection, but not OC43 infection, as shown in western blotting and progeny virus analysis (**Figures 5C, F**).

**Figure 6:**
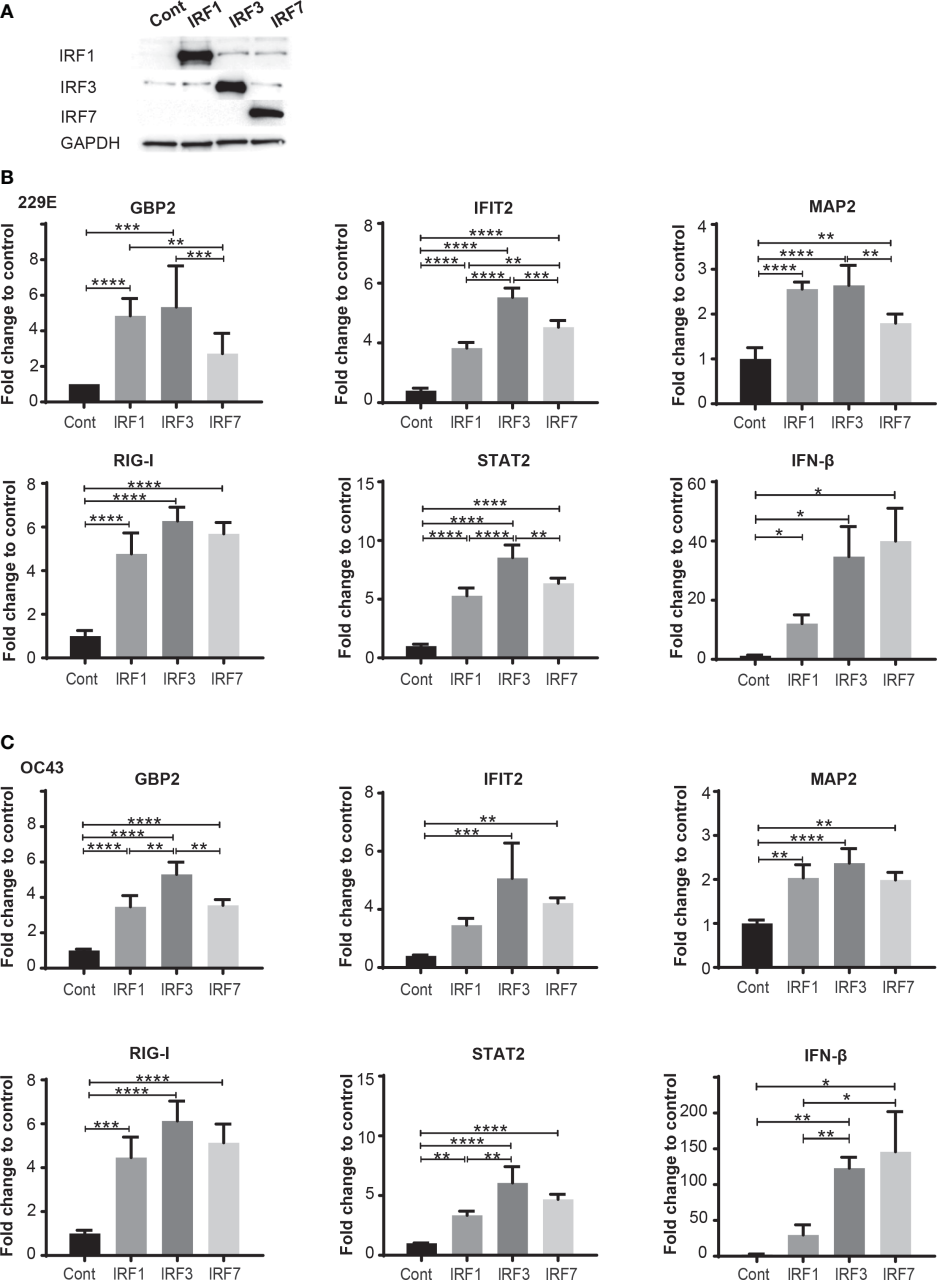
IRF3 effectively activates transcription of antiviral genes during human coronavirus infection. H1299 cells were transfected with control pcDNA3 plasmid (Cont) or the plasmid containing IRF1, IRF3 or IRF7 and then infected with or without 229E or OC43 at MOI of 0.01. **(A)** The expression of IRF1, IRF3 and IRF7 was confirmed by western blot analysis. At 24 hours after infection, the expression of GBP2, IFI2, MAP2, RIG-I, STAT2 and IFN-β in the cells infected with 229E **(B)** or OC43 **(C)** was determined by RT-qPCR (n=4). *p<0.05, **p<0.01, ***p<0.001, ****p<0.0001, Two-way ANOVA.

To clarify how IRFs demonstrate different antiviral effects against human coronavirus infection, we investigate expression levels of antiviral genes in cells transfected with control pcDNA3, IRF1-pcDNA3, IRF3-pcDNA3 or IRF7-pcDNA3 at 24 hours after infection of 229E or OC43 (**Figure 6**). First, the expressions of IRF1, IRF3 and IRF7 were confirmed (**Figure 6A**) and then the expression levels of the ISGs before infection were analyzed (**Supplementary Figure 5**). Following infection of 229E or OC43, most antiviral genes we examined (except GBP2 in IRF7-transfected cells infected with 229E and IFIT2 in IRF1-transfected cells infected with OC43) were significantly elevated in cells introduced with IRF1, IRF3 or IRF7 compared to the vector control infected cells (**Figures 6B, C**). Furthermore, the expression of GBP2, IFIT2, MAP2, STAT2 and IFN-β was higher in IRF3-transfected cells than in IRF1 and/or IRF7 transfected cells in response to 229E infection (**Figure 6B**). Similarly, GPB2 and STAT2 transcription were induced more in IRF3-transfected cells during OC43 infection (**Figure 6C**). These results suggest that IRF3 has the greatest ability to activate antiviral transcription during human coronavirus infection.

## Discussion

In this study, we demonstrated an antiviral role of the IFN-IRF axis against human coronavirus infection. We first found that human coronavirus 229E is moderately sensitive to type I and II IFN, while OC43 is not (**Figure 1**). Infection of both viruses efficiently induced ISGs and activated IRF1, IRF3 and IRF7, suggesting that the antiviral innate response of infected cells is not fully suppressed during infection (**Figures 2**, **3**). Activation of IRFs was also observed during SARS-CoV-2 infection (**Figure 3**). The loss- and gain-of-function experiments demonstrated that IRF1 and IRF3 have antiviral roles against OC43 infection, while IRF3 and IRF7 were effective in suppressing 229E infection (**Figures 4**, **5**).

We found that 229E is sensitive to type I and II IFN, in agreement with previous studies (20, 27). Type I IFN has been shown to inhibit OC43 infection in A549 (lung cancer cells), yet promotes it in NCI-H520 (lung cancer cells) or Huh 7.5 (hepatoma) (26). In our current study, IFN treatment did not have any effect on OC43 replication in MRC5 (normal fibroblast cells) and H1299 (lung cancer cells). This discrepancy could be due to the differences in cell types. Alternatively, it may be because of IFN concentrations used in this study, which are lower than those used in previous studies. The concentrations of IFN in the present study were based on our previous work on IFN-sensitive viruses (50, 62). A comparison in the same experimental system showed that the same concentration of IFN completely shuts down the replication of IFN-sensitive VSV, but only partially suppresses 229E while not affecting OC43 (**Figures 1F, G**). Thus, we conclude that the IFN sensitivity of human coronavirus 229E and OC43 is not very high.

Human coronaviruses are known to inhibit antiviral immunity induced by IFNs at various stages (63, 64). This was also evident in our study as OC43 infection was not sensitive to IFNs (**Figure 1**; **Supplementary Figure 1**). However, the ISGs were induced more efficiently in cells infected with OC43 than in those infected with 229E, which is relatively sensitive to IFNs (**Figure 2**). This may be because OC43 infection does not interfere with the ISG induction but may inhibit antiviral effectors involved at later stages of the IFN pathways such as protein kinase R (PKR), the 2′,5′-oligoadenylate synthetase (OAS)-RNase L pathway and Mx proteins. Moreover, we determined the expression levels of 6 selected ISGs among many ISG genes possibly induced during OC43 infection. Therefore, OC43 infection may suppress expression of other ISGs which may play major roles in protecting host cells. Lastly, induction of IFN-inducible transmembrane (IFITM), which is an effective antiviral protein for other viruses, promotes replication of OC43 (26), suggesting that ISG transcription may be required for its efficient replication.

Certain viral proteins of human coronaviruses are known to degrade IRF3 (44, 45). This was the case in our study where the expression of IRF3 was decreased during infection of 229E or OC43 (**Figure 3**). Nevertheless, we found that IRF3 was efficiently phosphorylated during viral infection (**Figure 3**). Furthermore, siRNA knockdown of IRF3 increased the susceptibility of host cells to 229E or OC43 infection (**Figure 4**). These results indicate that IRF3-mediated antiviral response is still active in cells infected with human coronaviruses, although viral evasion downregulates its expression. It was shown previously that BX795, which blocks phosphorylation and translocation of IRF3 (65), inhibits the induction of ISGs and promotes replication of SARS-CoV-2 (43). Considering that IRF3 overexpression inhibited 229E or OC43 infection (**Figure 5**) and promoted transcription of the antiviral genes more effectively than IRF1 or IRF7 (**Figure 6**), IRF3 may be a common antiviral effector against human coronavirus infection, which would make it an excellent antiviral therapeutic target. In contrast, the promotion of IRF1 showed antiviral activities in cells infected with OC43 while IRF7 expression reduced 229E infection (**Figure 5**). The expression analysis of the antiviral genes did not show the IRF1 and IRF7 bias between 229E and OC43 infection (**Figure 6**). To answer this, it is essential to further expand our study to investigate antiviral functions of the IRFs using global gene analysis and other human coronaviruses in the future.

IRF1, IRF3 and IRF7 are critical transcriptional regulators of IFNs and ISGs (19, 66, 67). Alternately, IFNs are major transcriptional activators of the IRFs (35). Thus, the activities of IFNs and IRFs are closely related. Interestingly, we found that OC43 is sensitive to antiviral effects of IRF1 and IRF3, but insensitive to IFNs. IRF1 and IRF3 have been reported to have antiviral functions independent of the IFN system, which may be essential to inhibit OC43 infection (41, 42, 68–70). IRF1 and IRF3 upregulate transcription of certain ISGs in IFN-independent manners during virus infection (41, 68–70). Moreover, IRF3 can establish antiviral responses in cells deficient in IFN production (42). Therefore, antiviral functions of IRF1 and IRF3, independent from the IFN system, may be involved in host antiviral responses against OC43 infection. This possibility warrants future investigation.

In our western blot analysis, we observed IRF1 bands, which are higher than expected, at 2 and 3 days after OC43 infection (**Figure 3B**). Interestingly, this shift was not observed in cells infected with 229E or SARS-CoV-2 infected cells. We believe that the size increase of IRF1 may be due to posttranslational modifications. The phosphorylation or monoubiquitination of IRF1 promotes its transcriptional activity (71). While this may be the reason why IRF1 showed antiviral activities against OC43 infection but not 229E infection (**Figure 5A**), it is yet to be clarified why they were observed only in cells infected with OC43.

We use MRC5 cells for most of our studies (**Figures 1**-**4**), but H1299 cells were used for the gain-of-function experiments for IRFs (**Figure 5**). This is because we encountered technical problems achieving sufficient expression levels of IRFs without causing cell morbidity or affecting virus infection in MRC5 cells during transfection.

## Data availability statement

The original contributions presented in the study are included in the article/**Supplementary Material**. Further inquiries can be directed to the corresponding author.

## Author contributions

JD: Conducted overexpression of IRFs, infection of the viruses and western blot analysis, and was involved in manuscript preparation and revising the manuscript. DX: conducted siRNA knockdown of IRFs, infection of the viruses, western blot analysis and RT-qPCR analysis, and involved in manuscript preparation. ML: conducted TCID50 assay, infection of viruses and western blot analysis and involved in manuscript preparation and revising the manuscript. MAJ and HAS: conducted SARS CoV-2 experiments. KL: Conducted tissue culture and western blot analysis. JG: Conducted SARS-CoV-2 experiments. DLT: Supervised the experiments conducted by MAJ and HAS. YK: Established the SARS-CoV-2 system and supervised the experiments conducted by JG. KH: Supervised the project, designed the experiments, and completed the manuscript. All authors contributed to the article and approved the submitted version.

## Supplementary material

Supplementary Figure 1 H1299 cells were left untreated or treated with IFN-α or IFN-γ, for 18 hours prior to infection (IFN prior to infection) or from 18 hours prior to infection to Day 6 (IFN whole period). The cells were challenged with 229E or OC43 infection at MOI of 0.01. **(A)** Western blot analysis of viral protein was conducted using anti 229E N protein, OC43 N protein and GAPDH antibodies. **(B)** TCID_50_ assay was performed to measure the progeny virus of 229E or OC43 at 6 days post infection. *p<0.05, **p<0.01, ***p<0.001, Two-way ANOVA.

Supplementary Figure 2
**(A)** MRC5 cells were left untreated or stimulated with IFN-α (500 U/ml) or IFN-γ (100 U/ml) for 18 hours. **(B, C)** MRC5 cells were left uninfected or infected with UV-inactivated 229E (B) or UV-inactivated OC43 **(C)** at MOI of 0.01 for 2 and 4 days. The expression levels of ISG mRNA (GBP2, RIG-I and STAT2) were determined by RT-qPCR. The relative quantification (RQ) indicates the fold change of the expression level for infected samples towards that of the non-infected controls at the same time point post infection. The transcriptional level for each gene was calculated by normalizing to GAPDH expression level and then normalized by the corresponding control. *p<0.05, **p<0.01, ***p<0.001, ****p<0.0001, Two-way ANOVA.

Supplementary Figure 3
**(A)** MRC5 cells were left untreated or stimulated with IFN-α (500 U/ml) or IFN-γ (100 U/ml) for 8, 16, 24 and 48 hours. **(B, C)** MRC5 cells were left uninfected or infected with UV-inactivated 229E **(B)** or UV-inactivated OC43 **(C)** at MOI of 0.01 for 2 and 4 days. The activation of IRF1, 3 and 7 was determined by western blot analysis using antibodies against 229E N protein, OC43 N protein, IRF1, IRF3, IRF7, phosphorylated (p-IRF3) and GAPDH.

Supplementary Figure 4 Human dermal fibroblasts were left uninfected (-) or infected (+) with 229E **(A)** or OC43 **(B)** at MOI of 0.01. The activation of IRF1, IRF3 and IRF7 was determined by western blot analysis using antibodies against 229E N protein, OC43 N protein, IRF1, IRF3, IRF7, phosphorylated (p-IRF3) and GAPDH.

Supplementary Figure 5 H1299 cells were transfected with control pcDNA3 plasmid (Cont) or the plasmid containing IRF1, IRF3 or IRF7. At 24 hours after transfection, the expression of GBP2, IFI2, MAP2, RIG-I, STAT2 and IFN-β was determined by RT-qPCR (n=4). *p<0.05, **p<0.01, ***p<0.001, ****p<0.0001, Two-way ANOVA.

Supplementary Table 1 Sequence of qPCR primers.
