# Supplementary figures and images for: Interferon regulatory factor 3 mediates effective antiviral responses to human coronavirus 229E and OC43 infection
Source: Front Immunol. 2023 May 1;14:930086. doi: 10.3389/fimmu.2023.930086 (PMC10183588; doi:10.3389/fimmu.2023.930086)

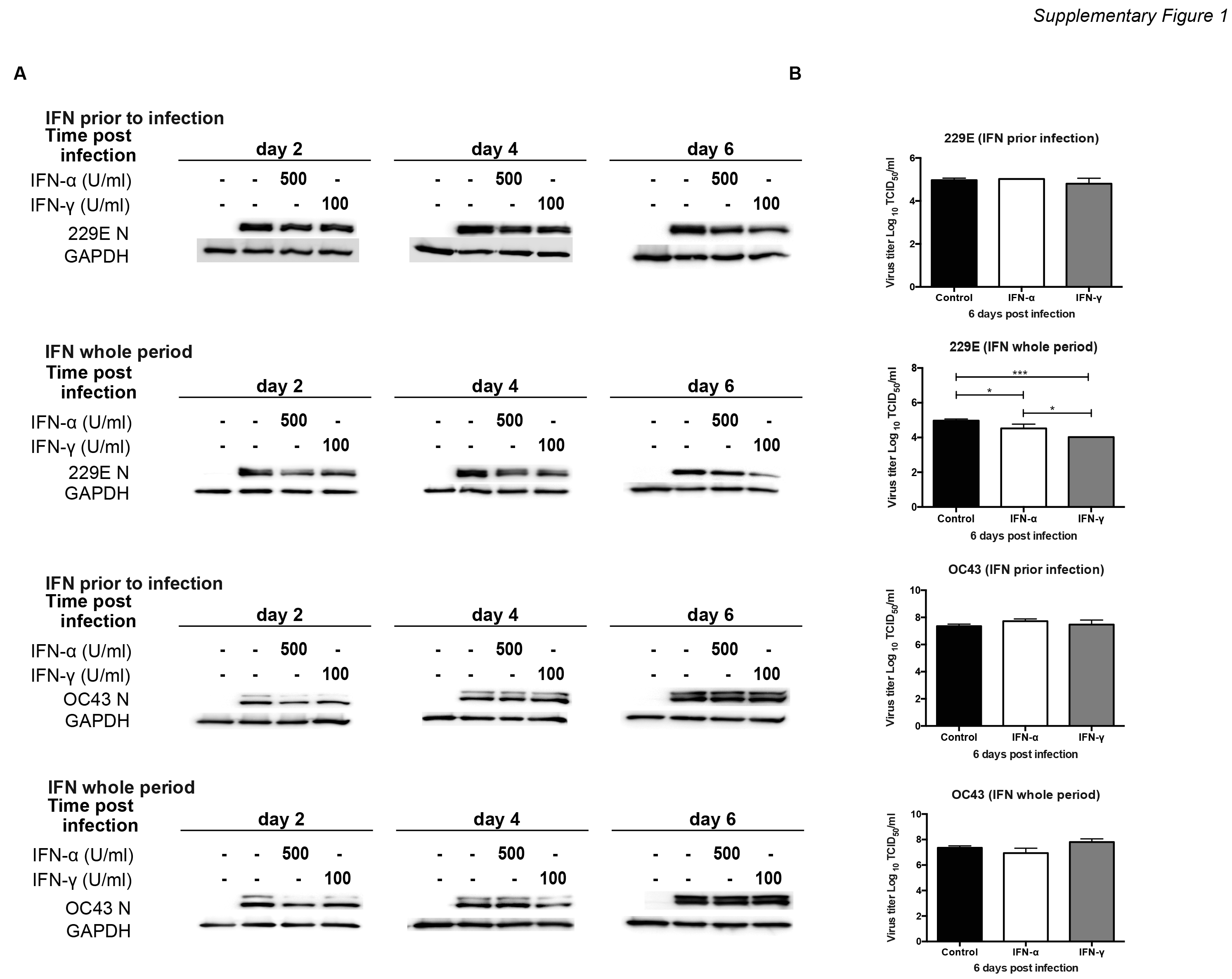

Supplement: Supplementary Figure 1 — H1299 cells were left untreated or treated with IFN-α or IFN-γ, for 18 hours prior to infection (IFN prior to infection) or from 18 hours prior to infection to Day 6 (IFN whole period). The cells were challenged with 229E or OC43 infection at MOI of 0.01. (A) Western blot analysis of viral protein was conducted using anti 229E N protein, OC43 N protein and GAPDH antibodies. (B) TCID50 assay was performed to measure the progeny virus of 229E or OC43 at 6 days post infection. *p<0.05, **p<0.01, ***p<0.001, Two-way ANOVA. [file Image_1.tif]

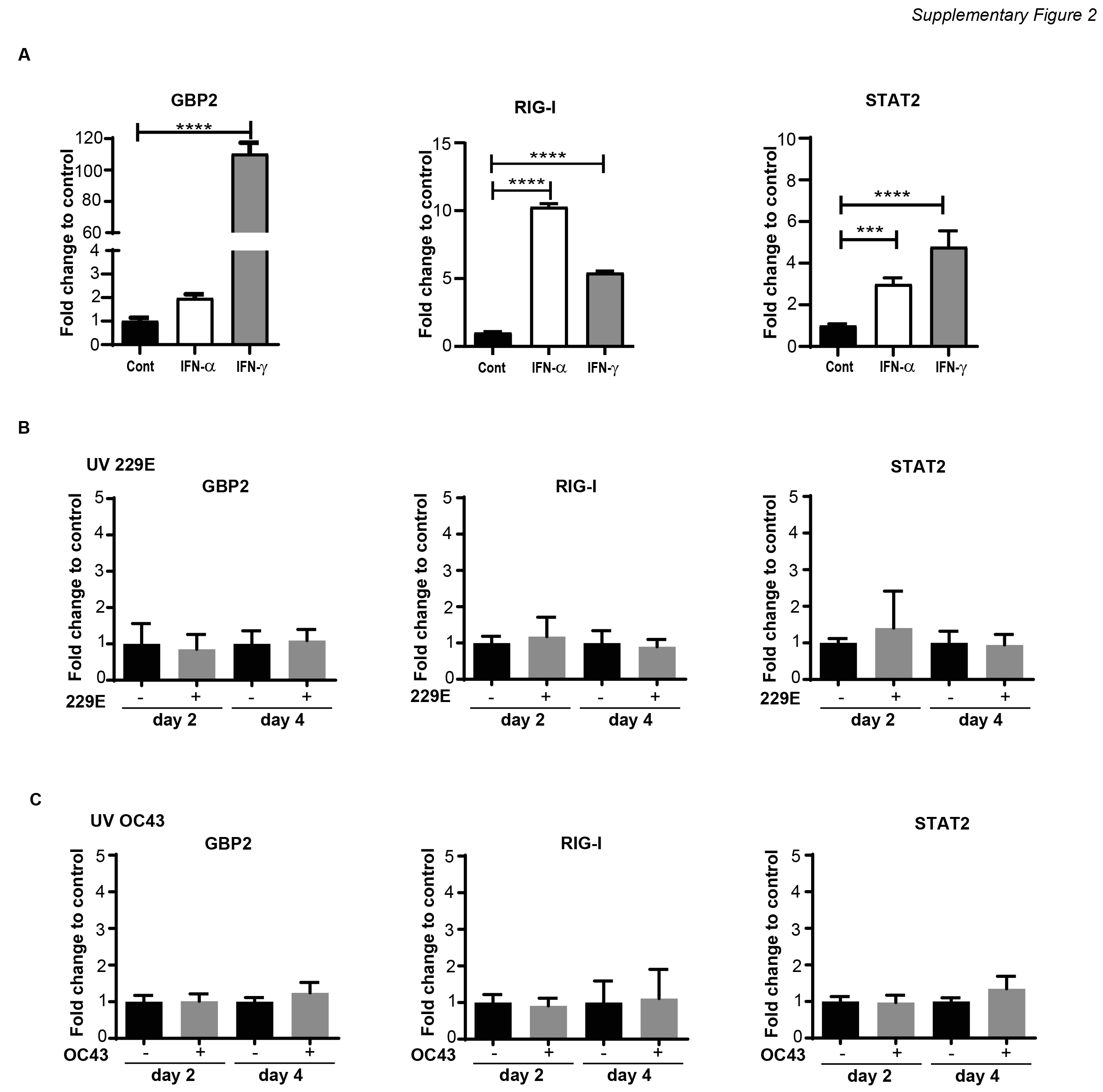

Supplement: Supplementary Figure 2 — (A) MRC5 cells were left untreated or stimulated with IFN-α (500 U/ml) or IFN-γ (100 U/ml) for 18 hours. (B, C) MRC5 cells were left uninfected or infected with UV-inactivated 229E (B) or UV-inactivated OC43 (C) at MOI of 0.01 for 2 and 4 days. The expression levels of ISG mRNA (GBP2, RIG-I and STAT2) were determined by RT-qPCR. The relative quantification (RQ) indicates the fold change of the expression level for infected samples towards that of the non-infected controls at the same time point post infection. The transcriptional level for each gene was calculated by normalizing to GAPDH expression level and then normalized by the corresponding control. *p<0.05, **p<0.01, ***p<0.001, ****p<0.0001, Two-way ANOVA. [file Image_2.tif]

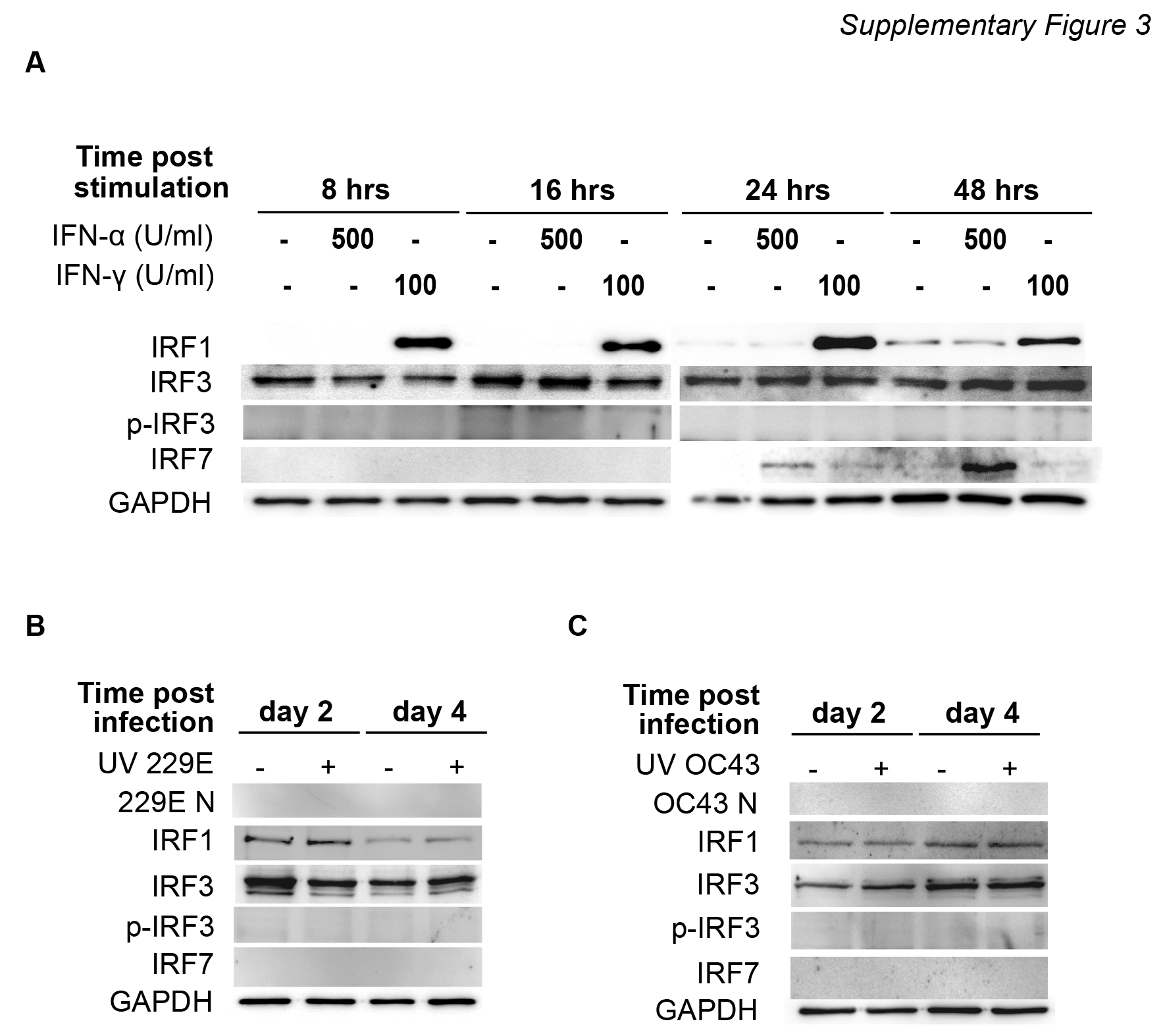

Supplement: Supplementary Figure 3 — (A) MRC5 cells were left untreated or stimulated with IFN-α (500 U/ml) or IFN-γ (100 U/ml) for 8, 16, 24 and 48 hours. (B, C) MRC5 cells were left uninfected or infected with UV-inactivated 229E (B) or UV-inactivated OC43 (C) at MOI of 0.01 for 2 and 4 days. The activation of IRF1, 3 and 7 was determined by western blot analysis using antibodies against 229E N protein, OC43 N protein, IRF1, IRF3, IRF7, phosphorylated (p-IRF3) and GAPDH. [file Image_3.tif]

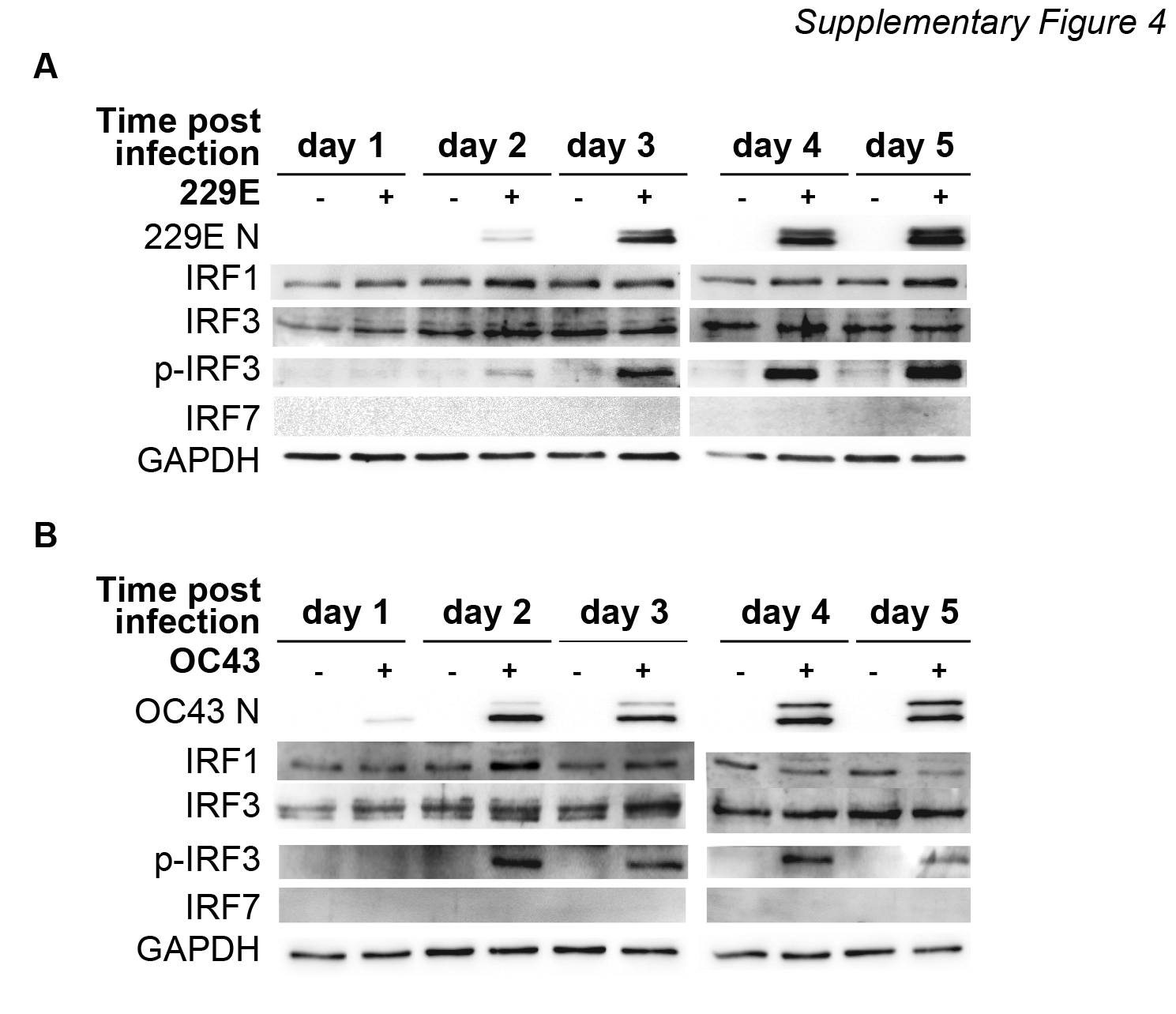

Supplement: Supplementary Figure 4 — Human dermal fibroblasts were left uninfected (-) or infected (+) with 229E (A) or OC43 (B) at MOI of 0.01. The activation of IRF1, IRF3 and IRF7 was determined by western blot analysis using antibodies against 229E N protein, OC43 N protein, IRF1, IRF3, IRF7, phosphorylated (p-IRF3) and GAPDH. [file Image_4.tif]

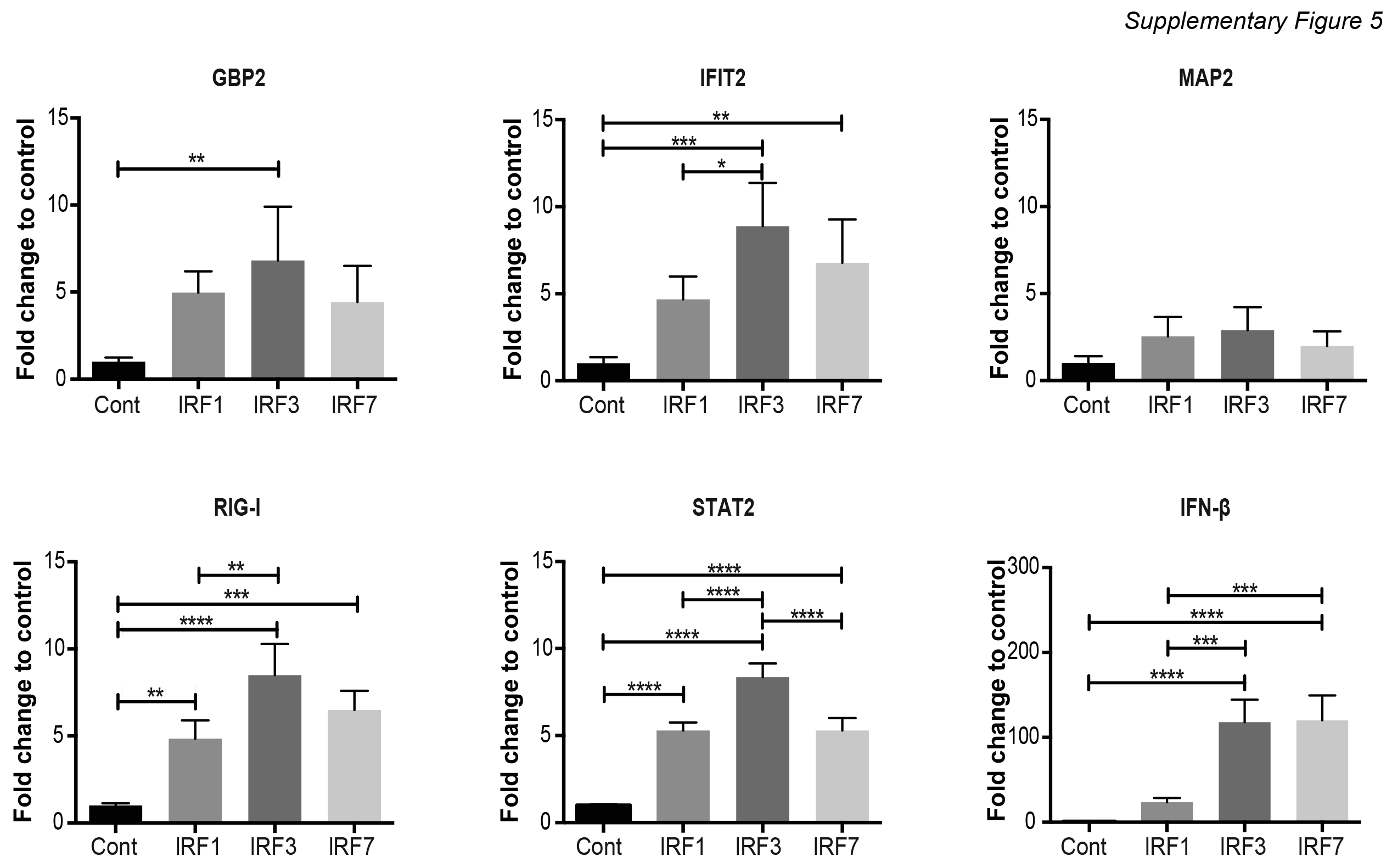

Supplement: Supplementary Figure 5 — H1299 cells were transfected with control pcDNA3 plasmid (Cont) or the plasmid containing IRF1, IRF3 or IRF7. At 24 hours after transfection, the expression of GBP2, IFI2, MAP2, RIG-I, STAT2 and IFN-β was determined by RT-qPCR (n=4). *p<0.05, **p<0.01, ***p<0.001, ****p<0.0001, Two-way ANOVA. [file Image_5.tif]
