# Supplementary material for: Interferon regulatory factor 3 mediates effective antiviral responses to human coronavirus 229E and OC43 infection
Source: Front Immunol. 2023 May 1;14:930086. doi: 10.3389/fimmu.2023.930086 (PMC10183588; doi:10.3389/fimmu.2023.930086)
Supplement: Supplementary Table 1 — Sequence of qPCR primers. [file Table_1.pdf]

Table S1. Sequence of qPCR primers

Gene name	5' primer	3' primer
229E N	GGCAAACGGGTGGATT TGTC	CGCCTAACACCGTAACC TGT
OC43 N	AGCAACCAGGCTGATG TCAATACC	AGCAGACCTTCCTGAGC CTTCAAT
GAPDH	ATCTTCTTTTGCGTCGC CAG	ACGACCAAATCCGTTGA CTCC
GBP2	TTTCACCCTGGAACTGG AAG	TGCACAACCGAGGATCA TTA
IFI44	TGAGGTCCAAGCTAGA GGAAGT	TTTACAGGGTCCAGCTC CCA
IFIT2	ATTGCACTGCAACCAT GAGTG	TCCCTCCATCAAGTTCCA GGT
IRF7	CCACGCTATACCATCTA CCT	TATCCAGGGAAGACACA CC
MAP2	GTTTCTGCGCCCAGATT TTATTG	TCCTCGGTTAGAGACAA GCTG
RIG-I	GCTGATGAAGGCATTG ACATTG	CAGCATTACTAGTCAGA AGGAAGC
STAT2	GGAATCAGGCATGTGT CCCTT	TTCACCTCTCACCCCAAT GGA
IFN- β	TGAGCAGTCTGCACCT GAAA	GCTTGAAGCAATTGTCC CGT
